# Targeted Enrichment of rRNA Gene Tandem Arrays for Ultra-Long Sequencing by Selective Restriction Endonuclease Digestion

**DOI:** 10.3389/fpls.2021.656049

**Published:** 2021-04-28

**Authors:** Anastasia McKinlay, Dalen Fultz, Feng Wang, Craig S. Pikaard

**Affiliations:** ^1^Department of Biology and Department of Molecular and Cellular Biochemistry, Indiana University, Bloomington, IN, United States; ^2^Howard Hughes Medical Institute, Indiana University, Bloomington, IN, United States

**Keywords:** Oxford Nanopore sequencing, *Arabidopsis thaliana*, ribosomal RNA gene enrichment, Nucleolus Organizer Region, NOR

## Abstract

Large regions of nearly identical repeats, such as the 45S ribosomal RNA (rRNA) genes of Nucleolus Organizer Regions (NORs), can account for major gaps in sequenced genomes. To assemble these regions, ultra-long sequencing reads that span multiple repeats have the potential to reveal sets of repeats that collectively have sufficient sequence variation to unambiguously define that interval and recognize overlapping reads. Because individual repetitive loci typically represent a small proportion of the genome, methods to enrich for the regions of interest are desirable. Here we describe a simple method that achieves greater than tenfold enrichment of *Arabidopsis thaliana* 45S rRNA gene sequences among ultra-long Oxford Nanopore Technology sequencing reads. This method employs agarose-embedded genomic DNA that is subjected to restriction endonucleases digestion using a cocktail of enzymes predicted to be non-cutters of rRNA genes. Most of the genome is digested into small fragments that diffuse out of the agar plugs, whereas rRNA gene arrays are retained. In principle, the approach can also be adapted for sequencing other repetitive loci for which gaps exist in a reference genome.

## Introduction

Many eukaryotic genomes have chromosomal loci that consist of hundreds, if not thousands, of nearly identical repeats, sometimes spanning millions of basepairs. Examples include the AT-rich satellites of pericentromeric regions (Aldrup-Macdonald and Sullivan, 2014), ribosomal RNA (rRNA) gene repeats (Gerbi, 1985; Flavell, 1986) and tandemly repeated transposable element (TE)-derived sequences (Ahmed and Liang, 2012). Distinguishing one repeat from the next can be difficult, precluding the easy determination of how individual repeats are arranged at the locus. As a result, repetitive loci are often miss-assembled or absent from assemblies of eukaryotic genomes (Biscotti et al., 2015).

Two long-read sequencing technologies have greatly improved the ability to close gaps in sequenced genomes, namely Pacific Biosciences (PacBio) SMRT sequencing and Oxford Nanopore MinION sequencing (Besser et al., 2018; Michael et al., 2018). PacBio sequencing can yield reads that are 10–100 kb in length, with the potential to obtain multiple reads of the same DNA fragment. This allows one to obtain consensus sequence reads whose accuracy rivals that of short-read Illumina or Sanger sequencing. By obtaining highly accurate long reads, single nucleotide polymorphisms (SNPs) can be identified among repeats that are nearly identical in sequence. For instance, PacBio sequencing has been used to identify subtle sequence differences among *Arabidopsis thaliana* rRNA genes (Havlova et al., 2016) that are each ∼10 kb in length. However, PacBio sequencing reads are not long enough for assembly of rRNA genes into long contigs due to the paucity of variation that is unique and thus not shared by numerous genes.

Sequencing using Oxford Nanopore Technology (ONT) yields ultra-long reads that can be hundreds of kilobases in length, but with an accuracy of only 75–95% (Rang et al., 2018). The technology is especially well-suited to identifying chromosomal deletions, insertions, or rearrangements. However, the high error rate of ONT sequencing is problematic for assembly of repetitive regions in which there are few sequence differences to discriminate each repeat (Michael et al., 2018). For successful assembly of these repetitive regions, having multiple overlapping ONT reads is necessary, thus allowing consensus sequences to be deduced to improve the accuracy and confidently identify SNPs and other subtle variation (Ebler et al., 2019).

Targeted enrichment aims to increase sequencing coverage for a region of interest (reviewed in Good, 2011; Kozarewa et al., 2015). Current methods are mostly designed for short-read sequencing, but some are amenable to ultra-long sequencing of large repetitive genomic regions. For instance, the clustered regularly interspaced short palindromic repeats (CRISPR) Cas9 system has been used for targeted sequencing (Bennett-Baker and Mueller, 2017; Gabrieli et al., 2018; Nachmanson et al., 2018). In this approach, megabase-sized genomic regions of interest are cleaved and purified from the rest of the genome by pulsed-field gel electrophoresis. Although compatible with PacBio and ONT sequencing, the strategy poses technical challenges and requires large amounts of starting DNA.

Nucleolus Organizer Regions (NORs) are missing from current genome assemblies of multicellular eukaryotes. The number of NORs in a genome varies between species, and within a species the number of rRNA genes within a NOR can vary between individuals and even among cells of an individual (Stults et al., 2008; Tucker et al., 2010; McStay, 2016). Due to the lack of substantial sequence variation among rRNA genes repeats, NORs of reference genomes are sometimes represented by a single rRNA gene repeat, with actual copy numbers and NOR sizes remaining unknown (Treangen and Salzberg, 2011). ONT sequencing holds promise for the assembly of NORs but has not yet been used to assemble complete NORs (Michael et al., 2018). This poses an obstacle to studies of NOR recombination, replication and repeat homogenization as well as studies of large-scale rRNA gene regulation. For instance, our laboratory is interested in understanding why the two NORs of the *Arabidopsis thaliana* strain Col-0 differ in expression, with one being constitutively active and the other falling silent during development (Chandrasekhara et al., 2016; Mohannath et al., 2016), an epigenetic phenomenon known as nucleolar dominance (McStay, 2006; Tucker et al., 2010). Each *Arabidopsis thaliana* NOR is composed of hundreds of tandemly repeated rRNA genes that are each ∼10 kb in length, such that both span several million basepairs of DNA (Copenhaver and Pikaard, 1996b). Evidence suggests that chromosomal context or position, rather than rRNA gene sequence variation, is responsible for the differential expression of the two NORs (Chandrasekhara et al., 2016; Mohannath et al., 2016), but the chromosomal basis for nucleolar dominance remains unknown. The possibility exists that one of more locus control elements might be embedded within the NORs, thus their complete sequence is desirable.

Here, we describe a simple method for enrichment of ultra-high molecular weight rRNA gene tandem arrays using a cocktail of restriction endonucleases predicted not cut an rRNA gene reference sequence. When used to treat genomic DNA embedded in an agarose plug, the enzymes digest most of the genome into small fragments that passively diffuse out of the agarose plug (Fritz and Musich, 1990). This depletes the plug of unwanted DNA fragments while retaining large DNA fragments that include rRNA gene arrays. Using *A. thaliana* rRNA genes as our example, the strategy yields a tenfold increase in ONT sequencing reads corresponding to rRNA genes. In principle, the method should also be adaptable for the enrichment of other target sequences, simply by altering the choice of restriction endonucleases.

## Materials and Methods

### Plant Material

*Arabidopsis thaliana* Col-0 plants (Arabidopsis Biological Resource Center stock #CS 70000) were surface-sterilized and grown on agar plates containing 0.5X Murashige and Skoog medium (MS). Plants were harvested after 2 weeks of growth under short-day conditions (8 h light, 16 h dark).

### Preparation of Genomic DNA

Ultra-high molecular weight DNA was purified from *Arabidopsis thaliana* Col-0 plants by following the Bionano Prep Plant Tissue DNA Isolation, Liquid Nitrogen Grinding Protocol (Bionano document number 30177)^1^ (summarized in Supplementary Figure 1). Briefly, 2 g of fresh tissue was placed in a pre-chilled (overnight at −80°C) mortar and ground in liquid nitrogen using a pre-chilled pestle then resuspended in 40 mL of ice-cold Bionano Prep Plant Tissue Homogenization Buffer (Part #20283) supplemented with 2-mercaptoethanol (0.2% final concentration) and 1 mM spermine-spermidine (known as Plant Tissue Homogenization Buffer *plus*). The suspension was passed sequentially through 100 μm (VWR Cat# 21008-950) and 40 μm cell strainers (VWR Cat# 21008-949) into a pre-chilled 50 mL conical tube on ice. Nuclei were then pelleted by centrifugation at 3,500 × g for 20 min at 4°C using a swinging bucket rotor. After discarding the supernatant, the pellet was resuspended in 1 mL of Plant Tissue Homogenization Buffer *plus* buffer with the assistance of a small paint brush that had been presoaked in the buffer. The resuspended nuclei were further diluted with 40 mL of ice-cold Plant Tissue Homogenization Buffer *plus* buffer and then subjected to centrifugation at 60 × g for 2 min at 4°C using a swinging bucket rotor to remove cell debris, with no braking during rotor deceleration. The supernatant was then subjected to another 40 μm filtration step (VWR Cat# 21008-949). Nuclei were collected by centrifugation at 3,500 × g for 20 min at 4°C using a swinging bucket rotor and washed three times by resuspension in in 30 mL of ice-cold Plant Tissue Homogenization Buffer *plus* and re-pelleting at 3,500 × g for 20 min at 4°C. The final nuclei pellet was resuspended in 3 mL of ice-cold Plant Tissue Homogenization Buffer *plus* and applied on top of the Density Gradient (Bionano Prep Density Gradient, catalog numbers 20281 and 20280). After centrifugation at 4,500 × g for 40 min at 4°C in a swinging bucket rotor, with no braking during deceleration, nuclei were recovered from the gradient and collected into a pre-chilled 15 mL conical tube on ice. Nuclei were then diluted with 14 mL of ice-cold Plant Tissue Homogenization Buffer *plus* and collected by centrifugation at 2,500 × g for 10 min at 4°C in a swinging bucket rotor, with no braking during deceleration. After carefully decanting the supernatant, nuclei were resuspended in 50 μL of ice-cold Density Gradient Buffer (Bionano Prep Density Gradient Buffer Cat #20280). The nuclei were then equilibrated to 43°C for 3 min and mixed with 30 μL of molten 2% agarose equilibrated at 43°C (CleanCut Low Melting Point, Bio-Rad, Cat# 1703594) using a wide-bore tip, and pipetted into a Bio-Rad CHEF Disposable Plug Mold (Bio-Rad, Cat# 170-3713). The final agarose concentration of the plugs was 0.82%. Plug molds were incubated at 4°C for 15 min to solidify the agarose.

Plugs containing embedded nuclei were then subjected to Proteinase K (20 mg/mL; 0.8 mg/plug; QIAGEN, Cat# 19131) and RNase A (100 μg/mL; 1 μg/plug; QIAGEN, Cat# 19101) digestion and washed according to the Bionano Prep Plant Tissue DNA Isolation, Liquid Nitrogen Grinding Protocol (document #30177). For rRNA gene enrichment, embedded nuclei were treated with a restriction endonuclease cocktail composed of six enzymes predicted to be rRNA gene non-cutters. Briefly, agarose plugs were placed in 50 mL conical tubes and were first incubated in 10 mL of T10E10 buffer (10 mM Tris-HCl, 10 mM EDTA, pH 8.0) supplemented with 2 mM PMSF for 1 h at 4°C. Plugs were then washed four times, 30 min each, at room temperature in 10 mL of T10E10 buffer without PMSF. Next, individual agarose plugs were washed twice, 1 h, at room temperature, with 1 ml of 1× restriction enzyme buffer [1× CutSmart buffer (NEB)]. After a second wash, the plug was incubated with 200 μL of 1× CutSmart buffer (NEB) containing 50 U each of the restriction endonucleases BstZ17I-HF (NEB #R3594), SpeI-HF (NEB #R3133), BclI-HF (NEB #R3160), SnaBI (NEB #R0130), MscI (NEB #R0534), and PvuII-HF (NEB #R3151) at 37°C overnight. The buffer was then removed and replaced with 500 μL of 20 mM Tris-HCl, 50 mM EDTA, pH 8.0 and incubated at 10 min at room to stop further digestion. The agarose plug was then subjected to 5 wash steps, each 15 min at room temperature, with 10 mL of TE buffer in order to deplete the plugs of short DNA digestion products fragments that can diffuse from the plugs, unlike large DNA fragments that are retained.

Plugs were then melted at 70°C for 2 min, equilibrated at 43°C for 5 min, and then incubated with 2 μL of 0.5 U/μL agarase (ThermoFisher Scientific, Cat# EO0461) per plug at 43°C for 45 min to digest the agar and liberate the encapsulated DNA. The resulting solution was then subjected to drop dialysis by applying genomic DNA on a 0.1μm dialysis membrane (Millipore, Cat# VCWP04700) floating on the surface of 15 mL of TE buffer inside a 6 cm petri dish. Dialysis was at room temperature for 45 min. DNA was assessed for quantity and quality using a Qubit dsDNA BR Assay kit and by agarose gel electrophoresis.

### Quantitative PCR (qPCR) Assay

Genomic DNA was subjected to electrophoresis using a 0.7% agarose gel in 0.5× TBE buffer for 1 h and 15 min at 100 V. The gel was then stained with GelRed diluted 1:10,000 in water (GoldBio # G-725-100). The intensively stained DNA band >23 kb, consisting of all DNA fragments greater than the resolving limit of the gel, was then excised and the DNA extracted using a QIAEX II Gel Extraction Kit (QIAGEN #20021). The resulting DNA was assessed by qPCR for the presence of 25S rRNA gene sequences and actin genes using the following forward (F) and reverse (R) primers:

25S_F: GAGTGCTTGAAATTGTCGGGAGGGAAG;

25S_R: CGAATCTTAGCGACAAAGGGCTGAATC;

actin_F: GAGAGATTCAGATGCCCAGAAGTC;

actin_R: TGGATTCCAGCAGCTTCCA.

### Oxford Nanopore MinION Sequencing and Analysis

DNA sequencing library preparation was performed using the Oxford Nanopore Rapid Library Kit (RAD-004). Sequencing was performed using a MinION sequencer with an R9.4.1 flow cell. Base-calling of raw ONT sequencing data was performed using Albacore v2.3.1. General FASTQ read statistics were calculated by NanoPlot (v 1.13). Length count distribution was analyzed using an R ggplot2 package. Statistical analysis was performed with GraphPad Prizm8 software. Percent sequence identity was calculated using minimap2 and the following Perl script (Li, 2018): <minimap2 -c reference.fasta query.fasta | perl -ane “if(/tp:A:P/&&/NM:i:(\d+)/){$n+ = $1; $m+ = $1 while/(\d+)M/g;$g+ = $1,++$o while/(\d+)[ID]/g} END{print(($n-$g+$o)/($m+$o),“\n”)}” >.

## Results

**FIGURE 1:**
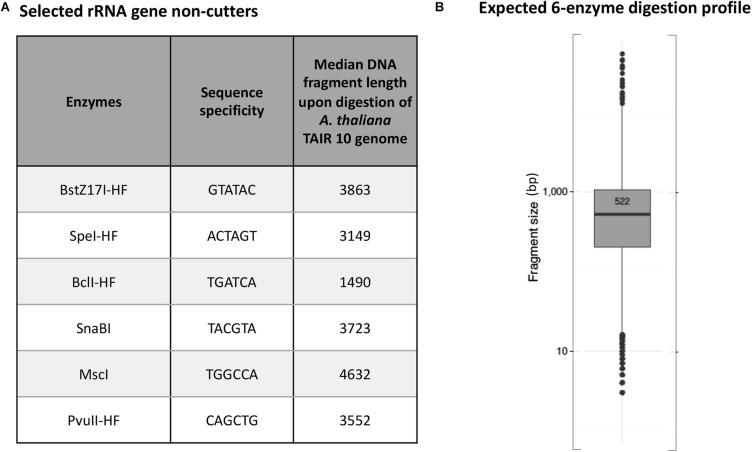
Predicted 45S rRNA gene non-cutting restriction endonucleases used as a cocktail for digestion of genomic DNA. **(A)** Names and sequence specificities of the enzymes and median fragment sizes obtained upon *in silico* digestion of *A. thaliana* Col-0 genomic DNA. **(B)** Expected *in silico* digestion mean size and distribution for Col-0 genomic DNA subjected to digestion by the six-enzyme cocktail.

*Arabidopsis thaliana* plants have two NORs that are located on the short arm of chromosomes 2 and 4 (Copenhaver et al., 1995; Copenhaver and Pikaard, 1996a). Each NOR is estimated to span ∼4 Mbp and consist of ∼350 to 400 rRNA genes that are each ∼10 kb in length (Tucker et al., 2010). We used the New England Biolabs (NEB) cutter tool (Vincze et al., 2003) to examine a full-length *Arabidopsis thaliana* (ecotype Col-0) 45S rRNA gene sequence (Chandrasekhara et al., 2016) and identify a list of 24 restriction endonucleases (RE) whose recognition sites are missing within the reference rRNA gene sequence. We then performed virtual *in silico* digestions of the *Arabidopsis thaliana* Col-0 reference genome (TAIR10) to identify the subset of these enzymes that cut most frequently in the genome (Supplementary Figures 2, 3), selecting six that are each predicted to digest genomic DNA to a median fragment length of 5 kb or smaller (Figure 1A) and that display 100% activity in NEB CutSmart buffer. *In silico* digestion using a cocktail of all six enzymes predicted that they would cut genomic *A. thaliana* (ecotype Col-0) into DNA fragments with a mean length of 522 bp (Figure 1B).

**FIGURE 2:**
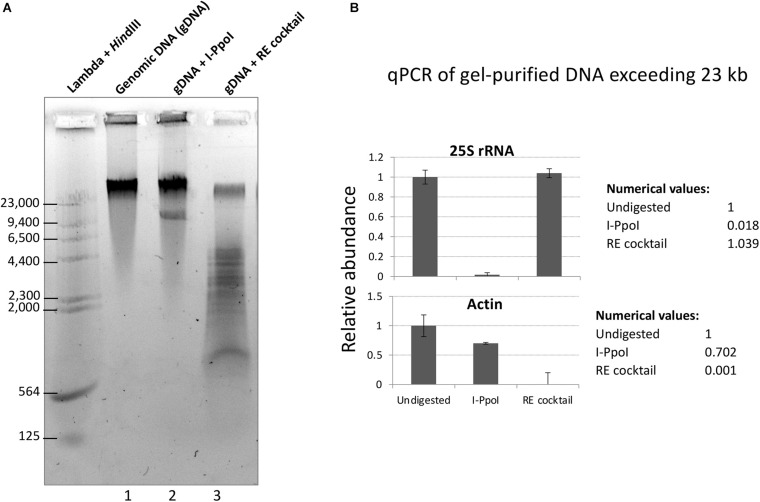
Digestion of genomic DNA with a cocktail of six restriction endonucleases (REs) predicted not to cut rRNA genes. **(A)** Col-0 gDNA that was either undigested (lane 1) or digested with I-PpoI (Lane 2) or the RE cocktail (lane 3) was separated on a 0.7% agarose gel and stained with GelRed dye. Bacteriophage Lambda DNA digested with *Hin*dIII provided size markers. **(B)** Summary of quantitative PCR (qPCR) results for rRNA gene (top panel) and actin (bottom panel) detection in undigested, I-PpoI, or RE cocktail-digested samples. qPCR was performed on gDNA extracted from the top gel band, containing DNA > 23 kb.

To test the restriction endonuclease cocktail, Col-0 genomic DNA immobilized in agarose plugs was subjected to in-plug digestion as described in Mohannath et al. (2016). Sizes of genomic DNA fragments were visualized following electrophoresis through a 0.7% agarose gel in TBE buffer. As shown in Figure 2A, digestion of genomic DNA with the enzyme cocktail resulted in a significant reduction of high molecular weight DNA (top band) and the appearance of DNA fragments mostly smaller than 5 kb (Figure 2A, lane 3). In contrast, treatment of genomic DNA with the rRNA gene-specific endonuclease I-PpoI, which cleaves once per rRNA gene (Muscarella et al., 1990; Copenhaver and Pikaard, 1996a), resulted in a band of ∼10 kb, the expected rRNA gene unit length (Figure 2A, lane 2). Quantitative PCR analysis of genomic DNA extracted from the top gel band revealed similar quantities of rRNA gene sequences in undigested genomic DNA and DNA cut by the six-enzyme cocktail. By contrast, digestion with I-PpoI depletes rRNA gene sequences, as expected (Figure 2B, top panel). The six-enzyme cocktail reduced the level of control actin gene DNA by 1,000-fold relative to undigested DNA (Figure 2B, bottom panel).

**FIGURE 3:**
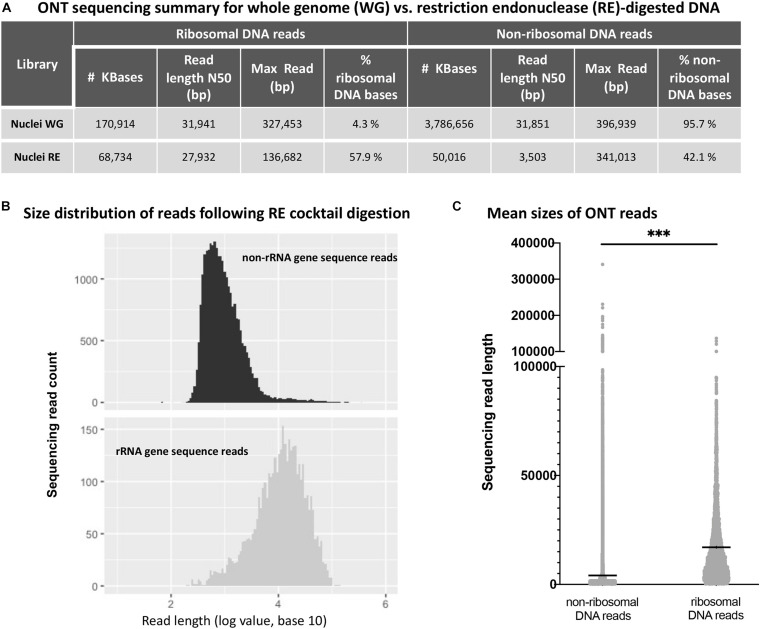
Oxford Nanopore sequencing results for whole genome (WG) vs. restriction endonuclease (RE)-digested DNA. **(A)** Effect of restriction endonuclease cocktail digestion on read distribution for non-ribosomal and ribosomal RNA gene sequences. **(B)** Non-ribosomal DNA (top) and ribosomal RNA gene DNA (bottom) read numbers are plotted as a function of read length. **(C)** Sequencing read mean length (horizontal black lines within the distributions) for non-ribosomal and ribosomal RNA gene sequences are significantly different (*** denotes unpaired *t*-test values of *p* < 0.0001).

Next, we performed ONT sequencing to test the degree to which digestion with the six-enzyme cocktail enriches for reads containing rRNA gene repeats. For this experiment, agarose-embedded nuclei from *Arabidopsis thaliana* Col-0 plants (denoted as whole genome, or WG nuclei in Figure 3A) were first subjected to digestion with Proteinase K and RNase A. Half of the sample was then incubated with the cocktail of six restriction endonucleases (RE nuclei) and the other half received only buffer. The resulting DNA was prepared via ONT’s rapid library kit (RAD-004) and sequenced on a MinION. Reads were mapped to the Col-0 reference genome (version TAIR10) with the alignment program ngmlr, using the default cutoffs (minimum identity of 65% and at least 25% of the read length aligned to the reference sequence) (Sedlazeck et al., 2018). The resulting FASTQ files (total sequenced DNA) were aligned to a rRNA gene consensus sequence (Chandrasekhara et al., 2016) in order to identify ribosomal gene DNA reads and separate them from remaining TAIR10-mappable sequences (non-ribosomal DNA reads). The sequence identity of the basecalled reads when aligned to the *A. thaliana* nuclear genome (TAIR10) was 85.78%. The sequence identity of the ribosomal gene reads was 86.12% for the 45S rRNA gene region (excluding the variable 3’ETS region). Sequencing statistics are shown in Figure 3A.

By definition, the N50 value of a sequencing run indicates a read length at which half of the total yield is in read lengths equal to or greater than this value. Consistent with targeted digestion of genomic DNA other than rRNA genes, the six-enzyme cocktail treatment greatly reduced the N50 read length for non-ribosomal DNA reads, whereas the N50 value for ribosomal DNA reads was less affected. Importantly, the percentage of sequenced rRNA gene bases (% ribosomal DNA bases) increased 13.5-fold, from 4.3% in the undigested control sample (Nuclei WG) to 57.9% in the sample digested with the six-enzyme cocktail (Nuclei RE). Additionally, the read length distribution of the restriction enzyme digested sample shows a statistically significant difference (unpaired *t*-test, *p*-value < 0.0001) between the non-ribosomal DNA reads and ribosomal RNA gene reads (Figure 3).

## Discussion

Gaps in published reference genomes can be millions of basepairs in size and can consist of repeats with nearly identical sequences, as is the case for NORs and pericentromeric repeats. Assembly of these regions can benefit from ultra-long ONT sequencing that yields reads that span multiple repeat units. However, a high depth of coverage is needed to assure accuracy and continuity of the assembly. Obtaining the needed coverage can be costly when the repeat region represents only a fraction of the genome to be sequenced.

In our proof-of-concept approach described in this brief report, we explored whether targeted enrichment of highly repetitive ribosomal RNA gene arrays can be combined with Oxford Nanopore Technology (ONT) sequencing in order to increase read depth coverage for *A. thaliana* NOR regions. Without enrichment, ribosomal RNA gene sequences are expected to account for ∼4.3% of the sequencing data, based on an estimated size of ∼8 Mbp for the two NORs (Copenhaver and Pikaard, 1996b). Restriction endonuclease-mediated sequence enrichment increased the proportion of rRNA gene reads by ∼13.5-fold. In our test, we used an RE cocktail chosen based on the sequence of a reference consensus gene sequence. A caveat to this approach is that rRNA gene sequence variants that can be cut by one or more of these enzymes may occur at low frequency. Thus, rRNA gene reads obtained by direct sequencing of genomic DNA, without restriction endonuclease digestion, should also be conducted. The latter can provide unbiased “scaffold reads” to which the “enriched” reads can be matched to increase the depth of sequence coverage. Alternatively, two or more different restriction endonuclease cocktails could be employed, designed to account for rare variants that might be cut using one cocktail but not another. These and other strategies for improving ultra-long sequencing coverage will likely be needed to achieve complete *de novo* assembly of NORs (Rang et al., 2018).

ONT sequencing of bacterial artificial chromosomes (BACs) is another way to obtain sequences for cloned arrays of tandem repeats, as recently demonstrated for BAC-cloned *Arabidopsis thaliana* rRNA gene arrays (Sims et al., 2021). An advantage of BACs is the ability to achieve high sequence coverage for the cloned insert, allowing high per-base accuracy. However, unlike direct genomic DNA sequencing, BACs tend to be ∼100 kb in size, which may not be long enough to identify sufficient variation for overlapping sequences to be identified and longer contigs assembled. BACs are also known to recombine, especially BACs containing cloned repetitive regions (Mozo et al., 1998). Thus, secondary confirmation of gene arrangements determined by BAC sequencing, obtained by direct genomic DNA sequencing to obtain even longer reads, is desirable and can have synergistic benefits, with ultra-long genomic DNA sequences serving as scaffolds for contig assembly and BAC sequences providing high accuracy at each nucleotide position within the contig.

Despite their overall conservation, 45S rRNA gene sequences are diverse in eukaryotes such that restriction endonuclease enrichment strategies must be adapted on a species-by-species, and even strain-by-strain, basis (Rabanal et al., 2017). However, the large selection of commercially available restriction enzymes makes it likely that the strategy be adapted for most species simply by altering the mix of restriction endonucleases. Exceptionally long read lengths will also likely be required to assemble NORs in species such as humans, in which individual rRNA gene repeats are four times longer (∼42 kb) than the ∼10 kb rRNA gene repeats of Arabidopsis. Thus, it is noteworthy that some of the longest reported ONT read lengths been obtained using human genomic DNA (Jain et al., 2018), far surpassing the ONT read lengths we have obtained thus far for Arabidopsis. Keeping these considerations in mind, if one has preliminary knowledge of repeat size and sequence variation, and the length of ONT reads possible for the species and cells being studied, enrichment strategies can likely be designed to obtain long reads to help assemble repetitive loci composed of highly similar genes or DNA elements.

## Data Availability Statement

The raw data supporting the conclusions of this article will be made available by the authors, without undue reservation.

## Author Contributions

CP conceived the experiments. AM designed and performed the experiments. FW performed *in silico* digestion of Col-0 gDNA by restriction enzymes. AM and DF analyzed results of ONT sequencing runs. AM and CP wrote the manuscript. All authors contributed to the article and approved the submitted version.

## Conflict of Interest

The authors declare that the research was conducted in the absence of any commercial or financial relationships that could be construed as a potential conflict of interest.

## Footnotes

1 https://bionanogenomics.com/wp-content/uploads/2018/02/30177-Bionano-Prep-Plant-Tissue-DNA-Isolation-Liquid-Nitrogen-Grinding-Protocol.pdf
