# Supplementary material for: Targeted Enrichment of rRNA Gene Tandem Arrays for Ultra-Long Sequencing by Selective Restriction Endonuclease Digestion
Source: Front Plant Sci. 2021 Apr 28;12:656049. doi: 10.3389/fpls.2021.656049 (PMC8113872; doi:10.3389/fpls.2021.656049)
Supplement: Supplementary file 1 [file Presentation_1.PPTX]

## Slide 1
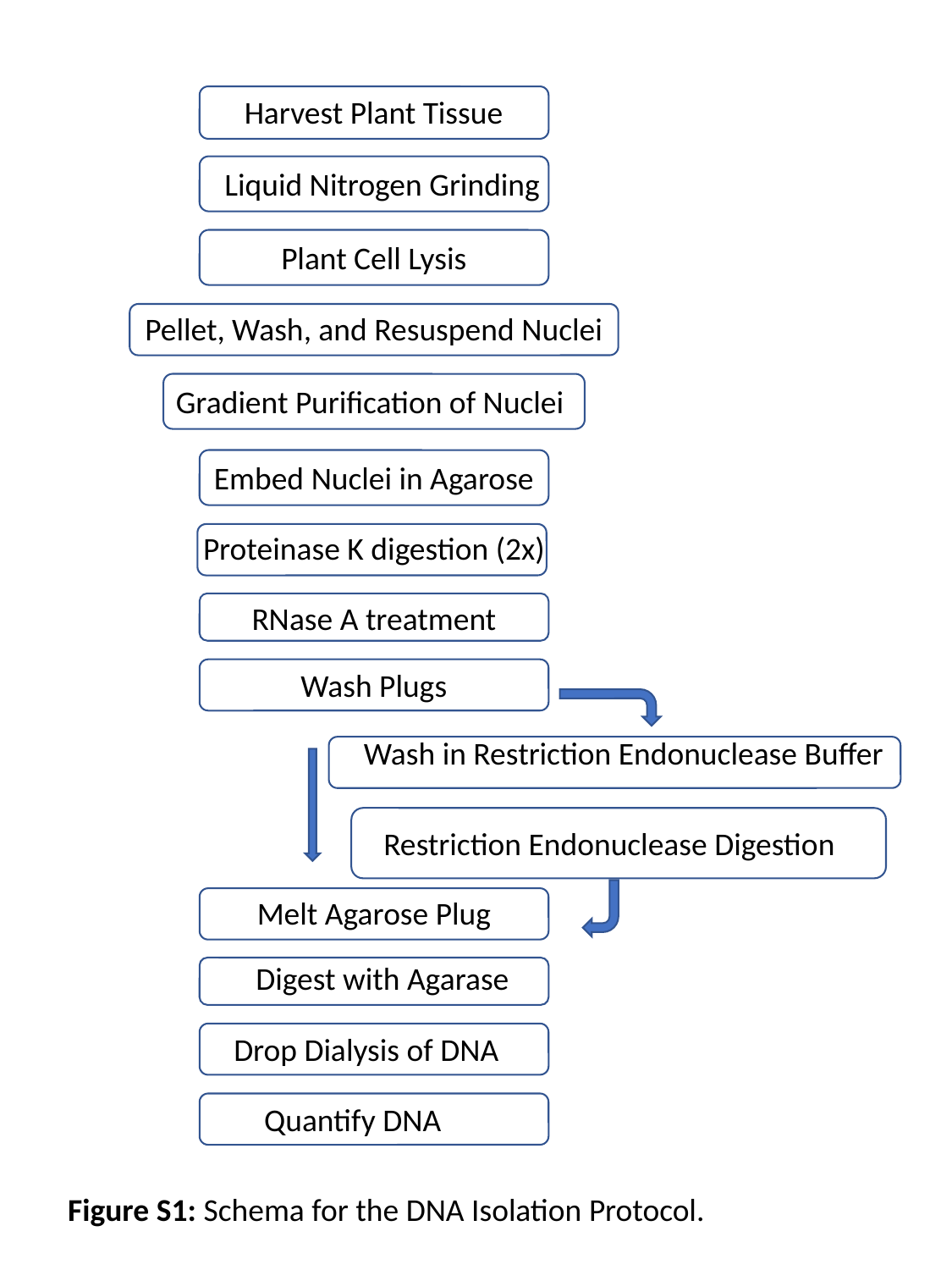

Harvest Plant Tissue
Liquid Nitrogen Grinding
Plant Cell Lysis
Pellet, Wash, and Resuspend Nuclei
Gradient Purification of Nuclei
Embed Nuclei in Agarose
Proteinase K digestion (2x)
RNase A treatment
Wash Plugs
Wash in Restriction Endonuclease Buffer
Restriction Endonuclease Digestion
Melt Agarose Plug
Digest with Agarase
Drop Dialysis of DNA
Quantify DNA
Figure S1: Schema for the DNA Isolation Protocol.

## Slide 2
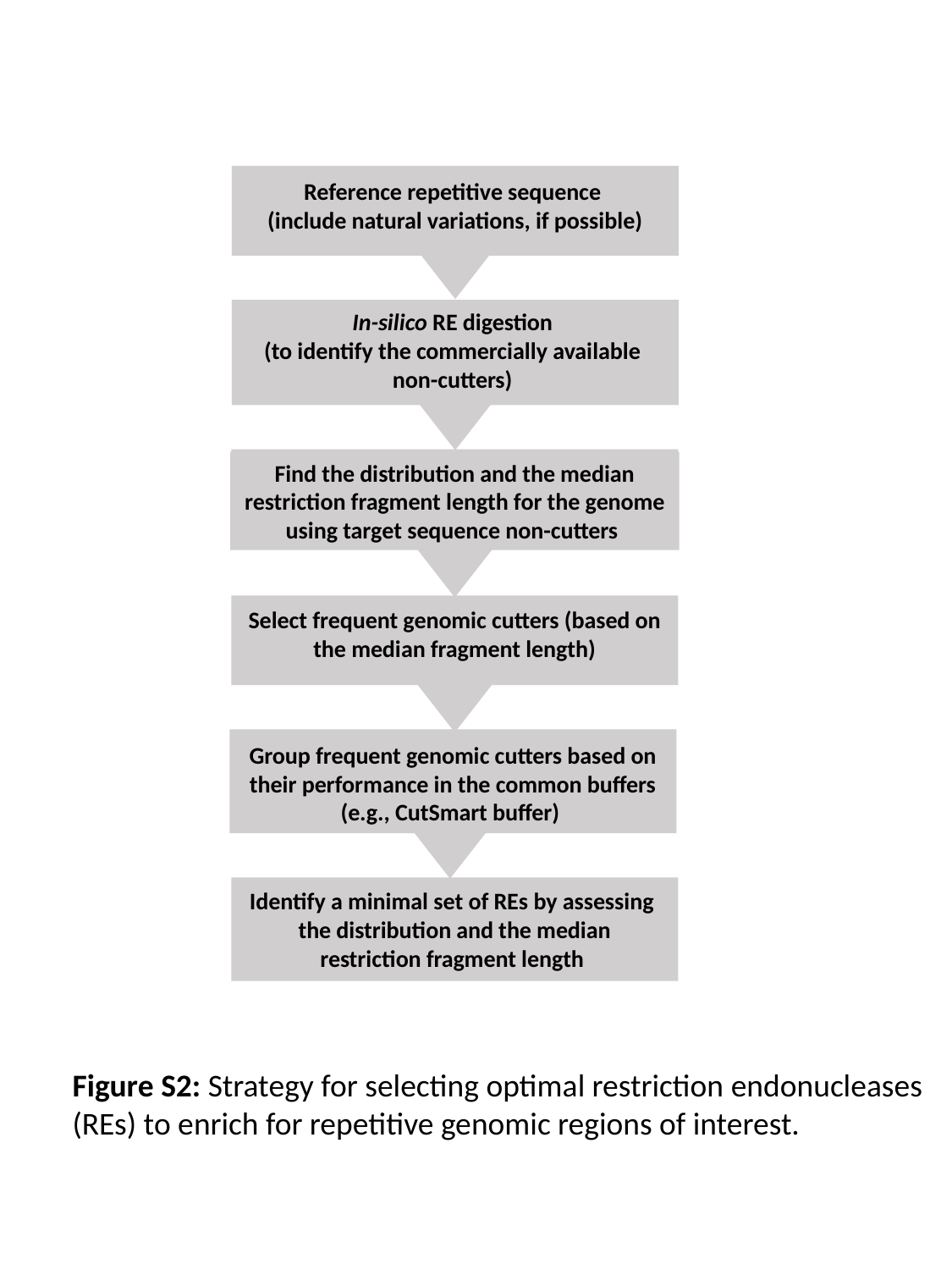

Reference repetitive sequence
(include natural variations, if possible)
In-silico RE digestion
(to identify the commercially available
non-cutters)
Find the distribution and the median restriction fragment length for the genome using target sequence non-cutters
Select frequent genomic cutters (based on the median fragment length)
Group frequent genomic cutters based on their performance in the common buffers
(e.g., CutSmart buffer)
Identify a minimal set of REs by assessing
the distribution and the median restriction fragment length
Figure S2: Strategy for selecting optimal restriction endonucleases
(REs) to enrich for repetitive genomic regions of interest.

## Slide 3
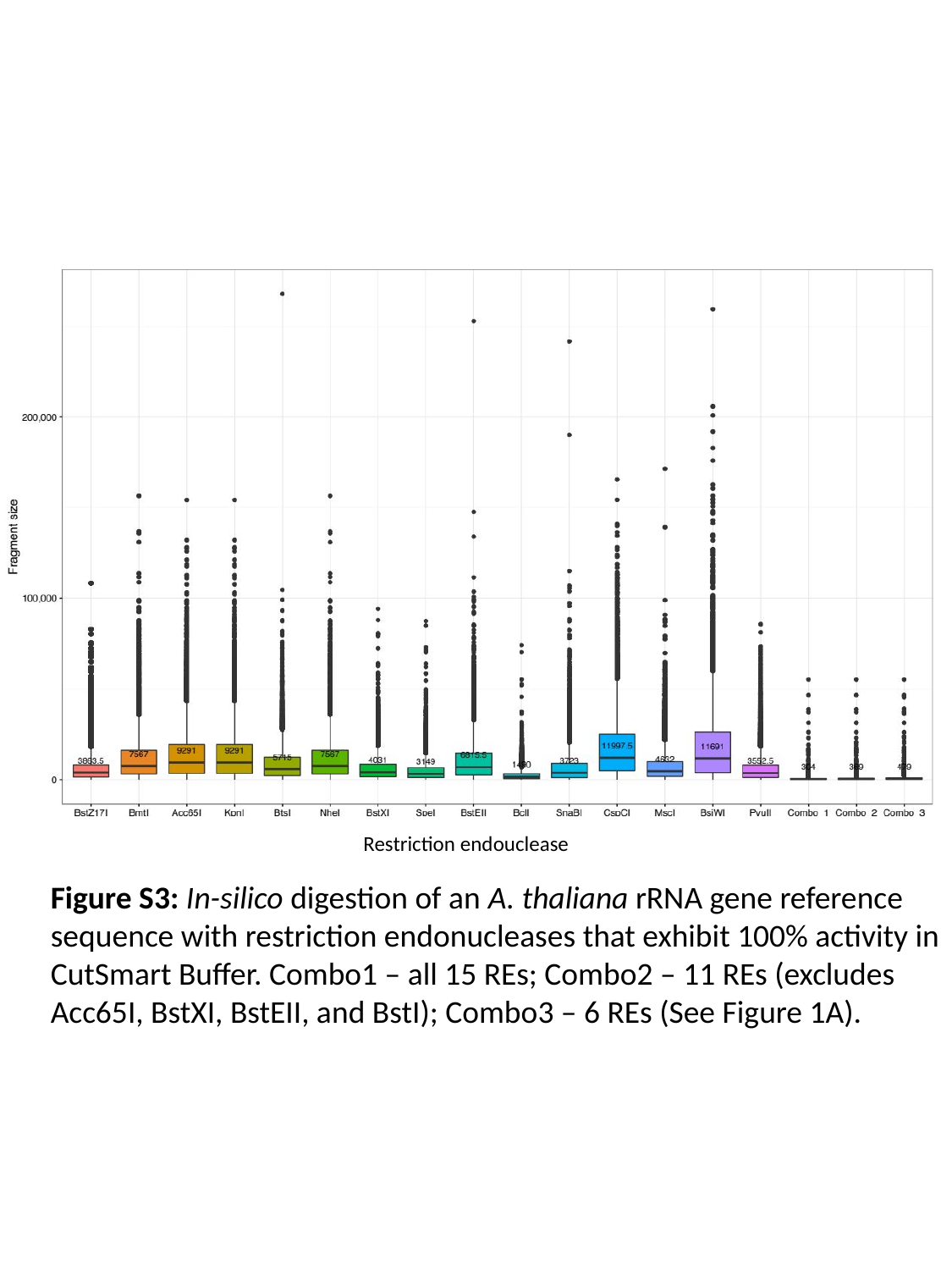

Restriction endouclease
Figure S3: In-silico digestion of an A. thaliana rRNA gene reference sequence with restriction endonucleases that exhibit 100% activity in CutSmart Buffer. Combo1 – all 15 REs; Combo2 – 11 REs (excludes Acc65I, BstXI, BstEII, and BstI); Combo3 – 6 REs (See Figure 1A).
